# The Potential and Challenges of Online Genetic Counseling in Japan

**DOI:** 10.31662/jmaj.2025-0533

**Published:** 2026-01-09

**Authors:** Yoshimitsu Fukushima

**Affiliations:** 1 Department of Medical Genetics and Department of Research Ethics and Integrity, Shinshu University School of Medicine

**Keywords:** Genetic counseling, Genetic counselor, Genomic Medicine Promotion Act, Online genetic counseling

In Japan, along with the rapid advancement of genetic medicine, the *Act on the Comprehensive and Systematic Promotion of Measures to Ensure that Citizens Can Receive High-Quality and Appropriate Genomic Medicine with Confidence* (abbreviated as the *Genomic Medicine Promotion Act*) was enacted in 2023 ^(1)^.

This law stipulates the need for appropriate consideration of bioethics and the development of consultation support systems; however, the term *“genetic counseling”* is not mentioned at all. Although the concept of genetic counseling is becoming widely recognized in society, who provides it and how it should be conducted remain undefined. Moreover, the main professionals responsible for genetic counseling―genetic counselors―are not yet nationally certified in Japan.

While awareness of the importance of genetic counseling has been increasing, its dissemination faces a serious challenge: a regional disparity caused by the concentration of specialists in urban areas and a shortage in rural regions.

Against this backdrop, the present study ^(2)^ systematically examined, for the first time, the clinical effectiveness of online genetic counseling (OGC) in Japan. The findings provide valuable insights into how Japan can enhance its genetic counseling service system and are therefore highly commendable.

The study found that both the OGC and in-person genetic counseling (IPGC) groups reported high satisfaction levels. Notably, the OGC group valued practical advantages such as time efficiency, reduced travel, and convenience. These results suggest that OGC could serve as a realistic solution to chronic issues in Japan’s healthcare system, including regional maldistribution of medical resources and limited accessibility for patients with mobility difficulties. Especially for individuals living in remote areas or those with visual impairments, the ability to receive expert support using devices suited to their own environment aligns with the philosophy of patient-centered care.

On the other hand, the study also highlighted areas where IPGC remains advantageous―namely nonverbal communication and emotional expression―indicating ongoing challenges in establishing trust and providing emotional support through virtual means.

Another key point is that technical and ethical issues such as privacy protection and network stability still constrain the broader adoption of OGC. In Japan, deep-seated cultural sensitivities surrounding genetic information and family relations may act as barriers to its implementation. Furthermore, the current limited insurance coverage creates economic and administrative burdens for both patients and healthcare providers, underscoring the urgent need for institutional support.

The limitations of the study include its single-institution design, small sample size, and narrow counseling focus. Nevertheless, as a pioneering effort to assess the feasibility of OGC in Japan, it deserves high recognition. Moving forward, multi-institutional collaborative research, comprehensive evaluations incorporating healthcare providers’ perspectives, and the development of culturally sensitive communication methods will be essential.

As healthcare continues to digitalize, Japan must strive to build a system that protects patient dignity and ensures access to professional support for all who need it. Moreover, the prompt national certification of genetic counselors is an urgent priority for the future of genomic medicine in Japan.

## Article Information

### Conflicts of Interest

None

### IRB Approval Code and Name of the Institution

None

### Acknowledgements

None
